# Supplementary material for: Insights of Fe2O3 and MoO3 Electrodes for Electrocatalytic CO2 Reduction in Aprotic Media
Source: Int J Mol Sci. 2022 Nov 2;23(21):13367. doi: 10.3390/ijms232113367 (PMC9658016; doi:10.3390/ijms232113367)
Supplement: Supplementary file 1 [file ijms-23-13367-s001.zip › ijms-1930044-supplementary.pdf]

Supplementary Materials

Insights of Fe_2O_3 and MoO_3 Electrodes for Electrocatalytic CO_2 Reduction in Aprotic Media

Néstor E. Mendieta-Reyes [1], Alejandra S. Lozano-Pérez [1] and Carlos A. Guerrero-Fajardo [1,*]

[1] Departamento de Química, Facultad de Ciencias , Universidad Nacional de Colombia, Bogotá 11321, Colombia
* Correspondence: caguerrerofa@unal.edu.co; Tel.: +57-3164308909

Corresponding Author

E-mail: caguerrerofa@unal.edu.co; Tel.: +57-3164308909

S1. Additional Experimental Details

Synthesis of thin films electrodes

For the preparation of Fe_2O_3 electrodes, the chemical bath deposit formation technique was used to grow nano-column films on a conductive FTO substrate. The experimental procedure consisted of adding 80.0 mL of an aqueous solution containing 0.15 M $FeCl_3$ $6H_2O$ (99%), and 1.0 M $NaNO_3$ (99%) in a flask where the FTO substrates are supported. The conditions of the chemical bath were 100 °C for 6 h. Subsequently, the thin films obtained were washed with deionized water to remove the salt residues from the precursors. Finally, the electrodes were subjected to an additional heat treatment in air at 600 °C for 1 hour to obtain the crystalline hematite phase.

The preparation of thin films of MoO_3 was carried out by the sol-gel method. First, a precursor solution was prepared by dissolving 3.0 g of MoO_3 (99.5%) in 20.0 mL of 30% hydrogen peroxide H_2O_2. The mixture was refluxed at 80 ° C for 2 h and then cooled to room temperature. The molybdenum oxide powders dissolved completely, and the solution turned yellow. The viscosity and concentration of the solution was adjusted with the addition of polyethylene glycol and 2-methoxyethanol under reflux for 30 min at 70 °C and it was allowed to cool to room temperature. After the reflux procedure with the additives, the solution remained yellow for several days maintaining an excess of H_2O_2. When the H_2O_2 concentration was not high enough, the solution turned dark blue due to the conversion of MoO_3 to $MoO_2(OH)(OOH)$.

S1.1. Analytical Procedures

Experiments were carried out in a closed and sealed glass cell with two compartments separated by a Nafion TM membrane NM-117. The Nafion membrane was prepared according to description of the reference [1]. Briefly Nafion membrane was first boiled in 3% (v/v) H_2O_2 and later in distilled water for 1 h, each one. Then the membrane was boiled in 0.5 M H_2SO_4 for 1 h, followed anew by boiling in distilled water for 1 h. Finally it was dried with a nitrogen flow before use in order to remove organic impurities. The working electrodes were a FTO thin film MoO_3/FTO and a Fe_2O_3/FTO electrode with 4 cm^2 of geometric area. A platinum wire was placed in the counter electrode compartment and an Ag/AgCl(s)/$KCl_{(aq)}$ was used as a reference electrode. Unless otherwise stated all the potentials were quoted versus this reference. Both compartments were filled with CO_2-purged 0.1 M TBAP ACN, and the cell was closed by sealing the joints with silicone. A potentiostatic electrolysis was made at −1.2 V 1.5 h in both cases. Samples of gas were extracted from the headspace of the working electrode compartment and analyzed with an Agilent 7820A gas chromatograph equipped with a thermal conductivity detector (Figure S1).

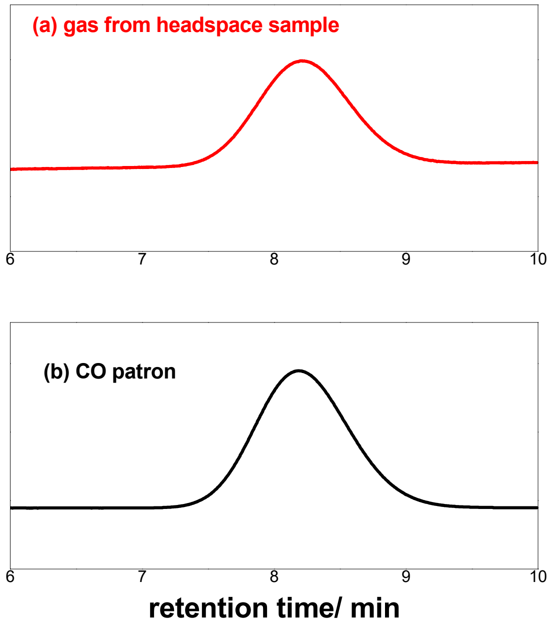

Figure S1. TPD-Gas chromatogram profiles for CO detection.

S2. Electrode Characterization

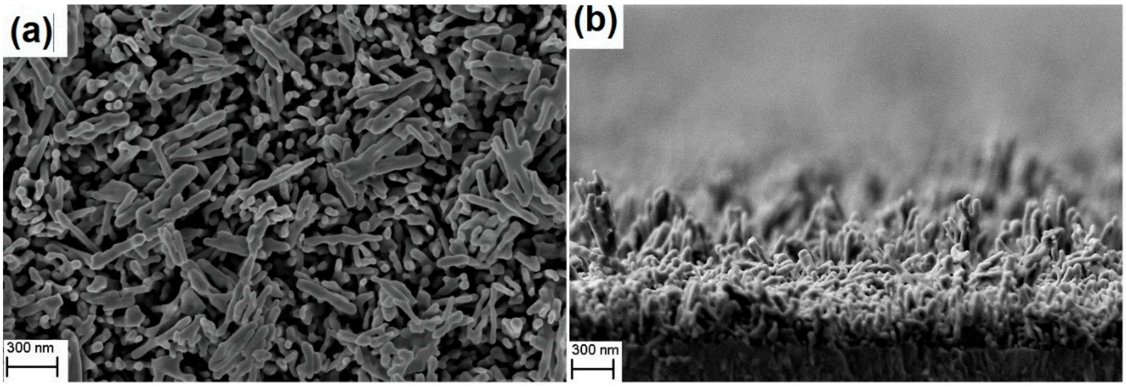

Figure S2. FE-SEM image of Fe_2O_3 nanostructured electrode surface obtained by chemical bath deposition over FTO substrate. (**a**) Top view; (**b**) electrode surface cross-sectional image.

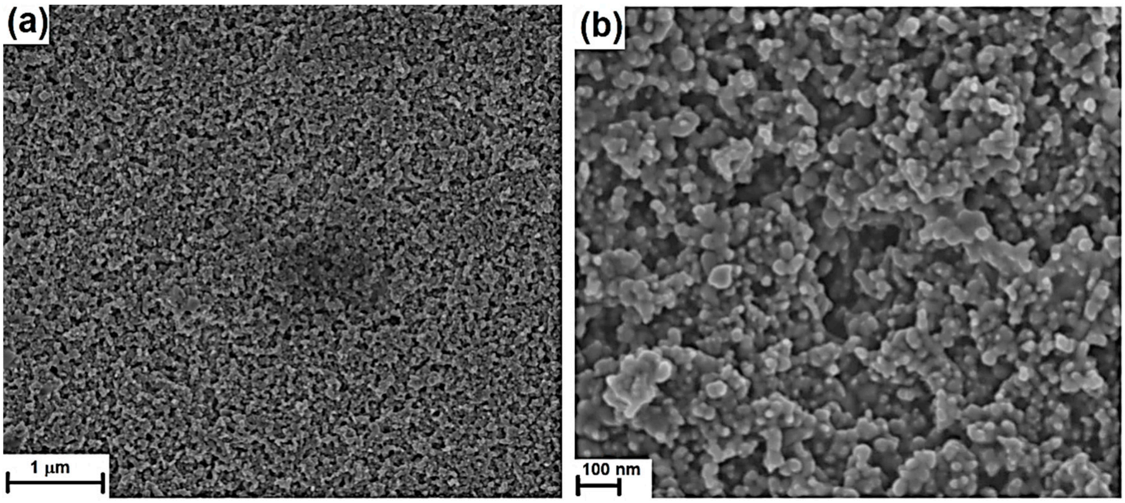

Figure S3. SEM images of MoO_3 nanostructured electrode surface deposited over FTO substrate. Spin-coated electrodes obtained from a MoO_3 precursor synthesized by sol-gel process. (**a**) 5000× and (**b**) 25,000× images.

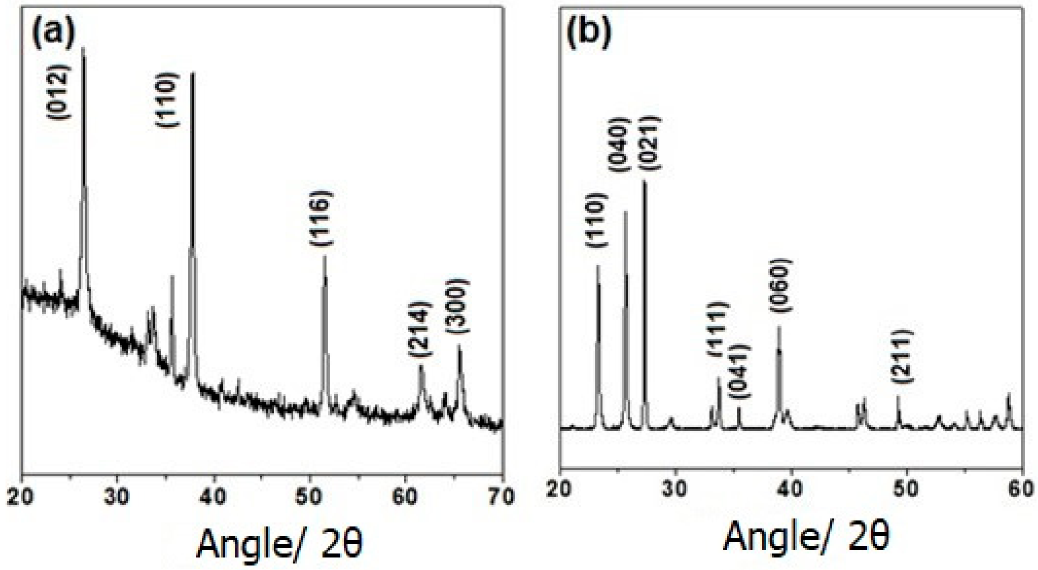

Figure S4. X-ray diffraction pattern of FTO coated thin film electrodes synthetized. (**a**) Fe_2O_3 and (**b**) MoO_3.

From the XRD pattern of an Fe_2O_3 electrode shown in Figure S4a, (110)-oriented hematite is evident. Peaks identification was made possible by comparison to JCPD reference values. XRD spectra shown in Figure S4b indicate well-defined peak positions that correspond to monoclinic MoO_3 phase.

S3. Flat Band Potential Determination

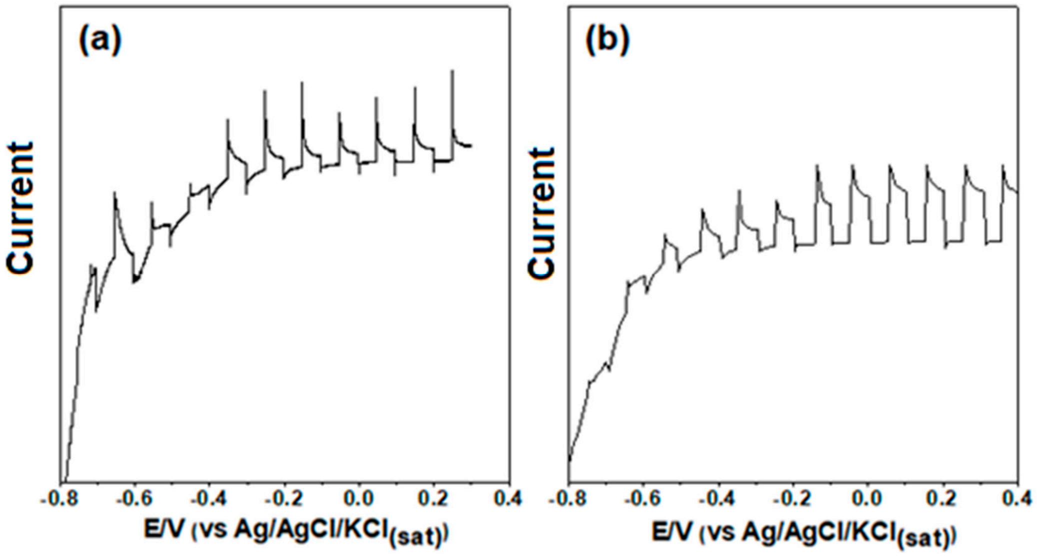

Figure S5. LSV voltammograms obtained at 5 mV·s^{-1} scanning rate for materials under Xe arc lamp illumination in 3.0 M methanol/0.1 M TBAF/ACN system purged with N_2. (**a**) Fe_2O_3 and (**b**) MoO_3 electrodes.

S4. Conversion of Potentials Measured to SHE Scale

A homemade Ag^+/Ag reference electrode was prepared using an Ag wire immersed in a glass tube filled with a 0.01 M $AgNO_3$ (97% from Sigma-Aldrich) + 0.1 M tetrabutylammonium perchlorate (≥99.0% from Sigma-Aldrich) in acetonitrile (99.8% anhydrous from Sigma-Aldrich) solution. Pavlishchuk and Addison have presented suitable conversion values for redox potentials measured against different reference electrodes in acetonitrile solutions, with a particular attention for conversions to the SHE scale [2]. In this way, the homemade reference electrode prepared by us is equivalent to the reference electrode reported by these authors as ANE3, whose conversion constant to the SHE scale is + 0.49 V. Therefore, we can convert the cathodic currents associated with CO_2 reduction onset potential measured against the Ag/Ag^+ electrode to the SHE scale, yielding a value of 0.11 V for Fe_2O_3 and −0.11 V for MoO_3

Supplementary References

(1) Hasani-Sadrabadi, M.M.; Dashtimoghadam, E.; Nasseri, R.; Karkhaneh, A.; Majedi, F.S.; Mokarram, N.; Renaud, P.; Jacob, K.I. Cellulose nanowhiskers to regulate the microstructure of perfluorosulfonate ionomers for high-performance fuel cells. *Journal of Materials Chemistry A* 2014, 2(29), 11334. https://doi.org/10.1039/c4ta00635f.

(2) Pavlishchuk, V.V.; Addison, A.W. Conversion constants for redox potentials measured versus different reference electrodes in acetonitrile solutions at 25 °C. *Inorganica Chimica Acta* 2000, 298(1), 97-102. https://doi.org/10.1016/s0020-1693(99)00407-7.
